# Assessing Microglial Dynamics by Live Imaging

**DOI:** 10.3389/fimmu.2021.617564

**Published:** 2021-03-08

**Authors:** Megumi Andoh, Ryuta Koyama

**Affiliations:** Laboratory of Chemical Pharmacology, Graduate School of Pharmaceutical Sciences, The University of Tokyo, Tokyo, Japan

**Keywords:** microglia, synapse, slice culture, acute slice, multiphoton/two-photon imaging, quadripartite synapse, microglial process, microglial motility

## Abstract

Microglia are highly dynamic in the brain in terms of their ability to migrate, proliferate, and phagocytose over the course of an individual's life. Real-time imaging is a useful tool to examine how microglial behavior is regulated and how it affects the surrounding environment. However, microglia are sensitive to environmental stimuli, so they possibly change their state during live imaging *in vivo*, mainly due to surgical damage, and *in vitro* due to various effects associated with culture conditions. Therefore, it is difficult to perform live imaging without compromising the properties of the microglia under physiological conditions. To overcome this barrier, various experimental conditions have been developed; recently, it has become possible to perform live imaging of so-called surveillant microglia *in vivo, ex vivo*, and *in vitro*, although there are various limitations. Now, we can choose *in vivo, ex vivo*, or *in vitro* live imaging systems according to the research objective. In this review, we discuss the advantages and disadvantages of each experimental system and outline the physiological significance and molecular mechanisms of microglial behavior that have been elucidated by live imaging.

## Introduction

Under physiological conditions, microglia have multiple finely branched protrusions, i.e., ramified processes, that constantly extend, and retract to monitor the extracellular environment (1–3). This dynamic process extension and retraction is characteristic of microglia, as neurons and astrocytes do not show significant morphological changes of their primary processes. These properties of microglia cannot be overlooked because they modulate microglial functions: production and directed release of inflammatory mediators, phagocytosis of pathogens and aggregate proteins, and cell–cell contacts (4). However, without a live imaging system, it is difficult to verify these phenomena; for example, if microglial density increases in a region as a result of observing fixed specimens, it is unclear whether this is due to microglial proliferation or migration. Additionally, it is difficult to discriminate whether increased cytokine expression or increased phagocytosis is the cause or effect of changes in the surrounding environment. Thus, understanding the molecular mechanisms and physiological significance of microglial motility requires direct live imaging of microglia while performing genetic and pharmacological manipulations.

Real-time imaging of microglia is essential; however, it is extremely difficult to observe microglia in their endogenous state in the brain. This is because microglia are sensitive to changes in the extracellular environment and can easily change their state during preparatory steps in experimental procedures, such as craniotomy (removal of the skull and replacement with a glass coverslip to allow transcranial viewing) for *in vivo* imaging, brain slice preparation for *ex vivo* imaging, and brain isolation for preparing cultures for *in vitro* imaging. More specifically, this may occur by surgical damage when performing live imaging *in vivo* and by various influences associated with culture conditions when performing live imaging *in vitro*, such as the presence or absence of other types of cells, the contents, serum, pH, and osmolality of the medium (5–8). Thus, if the microglia have already responded to the process of preparation in experiments, there is a possibility that further response of microglia by stimuli, including physical or pharmacological stimulation, may be underestimated, masked, or altered.

**Figure 1:**
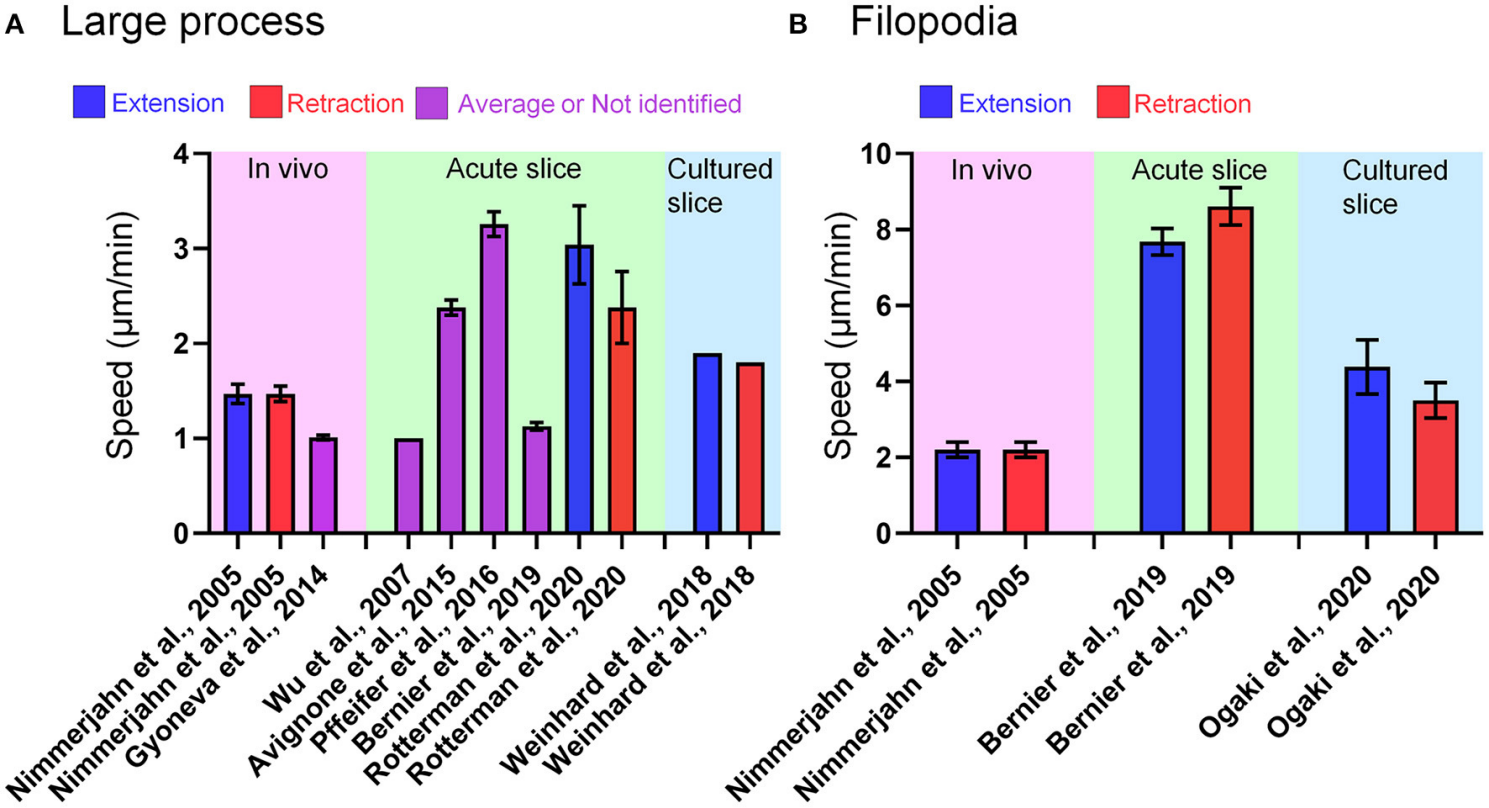
Microglial process motility in each live-imaging condition. **(A)** Speed of large process motility of microglia reported in papers referred in this review. In some cases, the speed of process motility was not specified as “extraction” or “retraction.” **(B)** Speed of filopodia motility of microglia reported in papers referred in this review. In Bernier et al. (9), the authors defined tiny protrusions near the tip of large process as filopodia, and provided the quantitative data of filopodia and large process. In Nimmerjahn et al. (1) and Ogaki et al. (10), short branches that emanated from the primary process of microglia were regarded as filopodia.

The spatiotemporal resolution of real-time imaging of microglia has improved considerably in recent years due to a variety of conditional investigations of observational techniques to overcome problems mentioned above. In this review, we highlight studies that performed live imaging of microglia *in vivo, ex vivo* (acutely prepared brain slices and organotypic brain slice cultures), and *in vitro* (dispersed culture of primary microglia). We further review the results of these studies, which are important for improving our understanding of microglial function and identity. In particular, it has been reported that the basic morphology and motility of microglial processes seen *in vivo* can be to some extent reproduced in acute slice and slice cultures (Figure 1). Therefore, in the following sections, observed phenomena (i.e., migration and phagocytosis), animal types (mouse, rat, and zebrafish), sample preparation methods (i.e., craniotomy, preparation of brain slices, and isolation of microglia from the brain), imaging conditions, and quantifiable parameters are compared to help microglial researchers interpret findings from live imaging studies and select appropriate methods for observing microglial dynamics.

## *In vivo* Live Imaging

### Essential Methods for *in vivo* Live Imaging

#### Preparation of Imaging Window

Mice and zebrafish have been often used for *in vivo* live imaging of microglia (Table 1). As the whole body including the brain of zebrafish is transparent, high-resolution imaging can be expected in any region if the sample is properly embedded and fixed in a gel. On the other hand, in case of mice, it is necessary to surgically create an imaging window because the skin and skull are not transparent enough for live imaging of the brain.

**Table 1:** Experimental conditions of live imaging of microglia *in vivo*.

**Animal**	**Age**	**Skull**	**Anesthesia**	**Region (depth from the** **surface)**	**Cellular visualization**	**Microscopy**	**Objective lens**	**Resolution (x, y) ** **z stack**	**Interval time ** **Total time**	**References**
CX3CR1-GFP^+/−^ mice	2 mo	Thinned	Isoflurane	Cortex	GFP (microglia)	Two-photon	25x (NA = 1.05)	– 1 μm step	1.5 min 13.5 min	Abiega et al. (11)
CX3CR1-GFP^+/−^ mice	8–12 mo	Open	Isoflurane	Cortex	GFP (microglia)	Two-photon	20x (NA = 1.0)	317 × 317 μm 50–80 μm with 1 μm step	– 22 days	Askew et al. (12)
CX3CR1-GFP^+/−^ mice	8–10 wo	Thinned	–	Cortex (100–150 μm)	GFP (microglia) mCherry (axonal bouton)	Two-photon	25x (NA = 1.10)	1,024 × 1,024 pixel (0.1269 μm/pixel) 1 μm step	1 min 3 h	Badimon et al. (13)
C57BL/6 mice	2–6 mo	Thinned	Isoflurane	Somatosensory cortex (~330 μm)	Twitch-2B (microglia)	Confocal	20x (NA = 1.0), 40x (NA = 0.8)	0.15–0.31 μm/pixel	1 frame/s, 10 min or 0.13 frame/s, 30–120 s	Brawek et al. (14)
pU.1::Gal4-UAS::TagRFP, mpeg1::Gal4-UAS::Kaede, nbt::DlexPR::NTR-mCherry, and slc7a7::Kaede (zebrafish)	2–3 dpf	–	0.01% tricaine		RFP, Kaede (microglia) mCherry (neuron)	Confocal	20x (NA = 0.4), 10x (NA = 0.25)	– 30–40 planes with 1.5–2 μm step	–	Casano et al. (15)
C57BL/6 mice	P4-19	Open	Isoflurane	Cortex (300–450 μm)	EGFP (neuron)	Two-photon	40×	78 × 79 μm 20 sections with 1 μm step	– ~2 h	Cruz-Martin et al. (16)
CX3CR1-GFP^+/−^;Thy1-YFP^+/−^ mice	–	Thinned	Ketamine and xylazine	Cortex (~300 μm)	GFP (microglia) YFP (neuron) SR101 (astrocyte) Hoechst (nuclei)	Two-photon	20x (NA = 1.0)	1,024 × 1,024 pixel (0.204 μm/pixel) 34 planes with 3 μm step	2–3 h –	Damisah et al. (17)
CX3CR1-GFP^+/−^ mice	–	Thinned	Ketamine and xylazine	Cortex (~200 μm)	GFP (microglia)	Two-photon	40x (NA = 0.8), 60x (NA = 0.9 or 1.0)	– 0.75–2 μm step	–	Davalos et al. (2)
CX3CR1-GFP^+/−^;Thy1-YFP^+/−^ mice	–	Thinned	–	Cortex	GFP (microglia) YFP (neuron) Alexa594 (fibrinogen)	Multi-photon	10x (NA = 0.4), 40x (NA = 0.8)	– 1.0–1.5 or 3–4 μm step	120–240 s 30–90 min	Davalos et al. (18)
CX3CR1-GFP^+/−^ mice	–	Open	Mixture of ketamine, xylazine and acepromazine	Spinal cord	GFP (microglia) rhodamine dextran (vessel)	Two-photon	–	–	–	Davalos et al. (19)
CX3CR1-GFP^+/−^ mice	P40-130	Open	Pentobarbital sodium and methohexital sodium	Spinal cord	GFP (microglia)	Two-photon	40x (NA = 0.75), 20x (NA = 1.0)	256 × 256 or 1,024 × 1,024 pixel (0.24–0.72 μm/pixel) 16–24 planes with 1.5–2.0 μm step	1–2 min –	Dibaj et al. (20)
WT mice CX3CR1-GFP^+/−^ mice	2–4 mo	Thinned	Isoflurane	Cortex	GFP, OGB-1, Fluo-4, Isolectin B4 conjugated to Alexa Fluor 594 (microglia)	Two-photon	40x (NA = 0.8), 60x (NA = 1.0)	– 10 μm with 1 μm step	–	Eichhoff et al. (21)
CD11b-CreERT2;R26-tdTomato;APPPS1 mice	4, 10 mo	Open	Isoflurane	Cortex (200–250 μm)	tdTomato (microglia)	Two-photon	25x (NA = 0.95)	0.27 μm/pixel or 1.49 μm/pixel –	Biweekly or monthly 1.5 years	Füger et al. (22)
TH-tdTomato^+/−^; CX3CR1-GFP^+/−^ mice	P56	Thinned	Isoflurane	Olfactory bulb	GFP (microglia) TH neuron (mCherry)	Two-photon	20x (NA = 1.0)	0.09 μm/pixel or 0.99 μm/pixel –	30 s 10 min	Grier et al. (23)
Thy1-YFP^+/−^ mice CX3CR1-EGFP^+/−^ mice	–	Thinned	Ketamine and xylazine	Cortex (~100 μm for neuron and ~200 μm for microglia)	YFP (neuron) EGFP (microglia)	Two-photon	60x (NA = 1.1)	512 × 512 pixel (66.7 × 66.7 μm) 0.75 μm step	– ~2 h	Grutzendler et al. (24)
Thy1-YFP^+/−^ mice (H-line)	6–7 mo	Open	Ketamine and xylazine	Hippocampus (50 μm below the pyramidal cell layer)	YFP (neuron)	Two-photon	16x (NA = 0.8)	100 × 100 μm (0.09 μm/pixel) 60 μm with 1 μm step	4 d interval –	Gu et al. (25)
CX3CR1-GFP^+/−^ mice	–	Thinned	–	Cortex	GFP (microglia)	Two-photon	-	– 15 planes with 2 μm step	4 min 40 min	Haynes et al. (26)
mpeg1:GFP (zebrafish)	4 dpf	–	0.01% MS-222	Brain (100–150 μm) trunk(80–120 μm)	GFP (microglia) PI (dead cell)	Confocal	-	– 3.6–6 μm step	6 min –	Herzog et al. (27)
CX3CR1-GFP^+/−^;Cnp-mEGFP^+/−^;Plp-DsRed^+/−^ mice	P30-1,100	Thinned or open	–	Somatosensory cortex (~75 μm)	GFP (microglia) mEGFP (myelin) DsRed (oligodendrocyte)	Confocal or two-photon	20x (NA = 1.0)	–	–	Hill et al. (28)
CX3CR1-GFP^+/−^ mice	6–10 wo	Thinned	Awake or mixture of ketamine and xylazine or pentobarbital	Somatosensory cortex (50–150 μm)	GFP (microglia)	Two-photon	20x (NA = 0.95)	521 × 521 pixel (0.38 μm/pixel) 26–37 planes with 1 μm step	30 s 15–20 min	Hristovska et al. (29)
NSG-CCR2-RFP^+/−^;CX3CR1-GFP^+/−^ mice	8–12 wo	–	Isoflurane	Frontal cortex	GFP (microglia) RFP (macrophage) BFP (tumor)	Two-photon	20x (NA = 0.95)	1,024 × 1,024 pixel (0.584 μm/pixel) 7 planes with 1 μm step	–	Hutter et al. (30)
Thy1-GFP^+/−^ mice (M-line)	–	Open or thinned	Ketamine and xylazine	Somatosensory cortex (~100 μm)	GFP (neuron)	Two-photon	25x (NA = 1.05)	78 × 78 μm (0.15 μm/pixel) 0.75 or 0.5 μm step	90 min –	Isshiki and Okabe (31)
Tg(Apo-E:eGFP) (zebrafish)	5–8 dpf	–	Awake	–	GFP (microglia) OGB-AM (Ca^2+^)	Confocal or two-photon	40x (NA = 0.80)	– 1 μm step	2–4 s or 1.5–5 min	Li et al. (32)
CX3CR1-GFP^+/−^ mice	2–3 mo	Open	Awake or isoflurane or ketamine and xylazine	Cortex	GFP (microglia)	Two-photon	40x	512 × 512 pixel (0.35 μm/pixel) 8 planes with 2 μm step	1 min –	Liu et al. (33)
Iba1-EGFP^+/−^ mice	P8-10	Open	Urethane and atropine or awake	Cortex (45–250 μm)	GFP (microglia) tdTomato (neuron) GCaMP6m (Ca^2+^)	Two-photon	25x (NA = 1.05)	512 × 512 pixel (0.099 μm/pixel) 0.5 μm step	5 min, 30 min-2 h or 1.6 s-1 min, 27 min or 8 Hz, 30 min	Miyamoto et al. (34)
Tg(mpeg1:GAL4, UAS:mCherry); rwTg(isl1:GFP); Tg(-3.5ubb:secAnnexin V-mVenus) (zebrafish)	2–5 dpf	–	0.01% tricaine	Spinal cord	mCherry (microglia) GFP (neuron) mVenus (AnnexinV)	Confocal	10x (NA = 0.3), 40x (NA = 0.8), 63x (NA = 0.9)	– 10–15 planes with 1–2 μm step	3–8 min	Morsch et al. (35)
CX3CR1-GFP^+/−^ mice	1.5–15 mo	Thinned	Isoflurane	Cortex (75 μm)	GFP (microglia)	Two-photon	–	– 15–25 planes with 1–2 μm step	20–45 s Several hours	Nimmerjahn et al. (1)
CX3CR1-GFP^+/−^ mice	2–4, 9–11, 18–21 mo	Thinned	Isoflurane	Cortex (80–100 μm)	GFP (microglia) Oregon Green 488 (Ca^2+^)	Two-photon	40x (NA = 0.80)	–	4 frames/s or 10 frames/s or 30 s, 20 min	Olmedillas Del Moral et al. (36)
CX3CR1^CreER/+^: Thy1 YFP-H mice	P19-34	Thinned	Ketamine and xylazine	Motor cortex (~100 μm)	YFP (neuron)	Two-photon	60x (NA = 1.1)	–	4 d –	Parkhurst et al. (37)
ApoE-GFP;NBT-DsRed (zebrafish)	3 dpf	–	0.01% tricaine	–	GFP (microglia) DsRed (neuron) LysoTracker DND-9 (lysosome)	Confocal	40x (NA = 1.2)	– 4 stacks spanning 10 μm	–	Peri et al. (38)
PC::G5-tdTomato mice	2–4 mo	Thinned	Isoflurane	Visual or somatosensory cortex (~100 μm)	tdTomato (microglia) GCaMP5G (Ca^2+^)	Two-photon	16x (NA = 0.8)	512 × 512 or 1,024 × 1,024 pixel	0.125 frames/s or 0.5 Hz –	Pozner et al. (39)
B6.Cg-Tg(Thy1-YFP)HJrs/J mice (#003782, JaxLab)	4–10 wo	Open	Isoflurane and ketamine	Somatosensory cortex	YFP (neuron)	Two-photon	25x (NA = 1.05)	166.7 × 166.7 μm (512 × 512 or 800 × 800 pixel) 30–70 μm with 1 μm step	24 h –	Pryazhnikov et al. (40)
CX3CR1-GFP^+/−^;Thy1-YFP^+/−^ mice	3–17 wo	Open	Awake or dexmedetomidine and midazolam	Cortex	GFP (microglia) YFP (neuron)	Two-photon	20x (NA = 0.95)	– 1 μm step	5 min 1 h	Stowell et al. (41)
CX3CR1-GFP^+/−^ mice	3–4 mo	Open	Awake or isoflurane or ketamine	Cortex (100–150 μm)	GFP (microglia)	Two-photon	20x (NA = 1.00)	512 × 512 pixel (0.77 μm/pixel) 6 planes with 2 μm step	20 s 33 min	Sun et al. (42)
CX3CR1-GFP^+/−^ mice, CX3CR1CreER-eYFP mice, and Rosa-CAG-LSL-eYFP-WPRE mice	2–5 mo	Open	Isoflurane	Somatosensory cortex (50–100 μm)	GFP, YFP (microglia) Evans lue,Rhodamine-dextran (vessel)	Multi-photon	40x (NA = 0.8)	800 × 800 pixel (0.18 μm/pixel) –	4 min 40–60 min	Taylor et al. (43)
CX3CR1-GFP^+/−^;Thy1-YFP^+/−^ mice	P28-39	Thinned	–	Primary visual cortex (50 μm)	GFP (microglia) YFP (neuron)	Two-photon	20x (NA = 0.95)	– 1 μm step	5 min 30 min-2 h	Tremblay et al. (44)
Rosa26-CAG-LSL-GCaMP6s mice, Rosa26-CAG-LSL-Lck-GCaMP6f mice, and CX3CR1^CreER−eYFP^ mice	3–5 mo	Open	Awake	Somatosensory cortex (55–80 μm)	GCaMP6s, GCaMP6f (Ca^2+^) GFP (microglia)	Two-photon	16x (NA = 0.8)	512 × 512 pixel (300 × 300 μm)	1 s –	Umpierre et al. (45)
Iba1-EGFP^+/−^;Thy1-GFP^+/−^ mice	6–10 wo	Thinned	Ketamine and xylazine	Cortex (100–250 μm)	GFP (microglia) YFP (neuron)	Two-photon	60x (NA = 1.1)	– 40–50 planes with 0.5 μm step	0.3–1.0 s –	Wake et al. (3)
Thy1-YFP^+/−^ mice (H-line)	2–7 mo	Open or thinned	–	Barrel cortex (0–100 μm)	YFP (neuron)	Two-photon	60x (NA = 0.9)	–	–	Xu et al. (5)
Tg(-2.8elavl3:eGFP; coro1a:DsRedx) (zebrafish)	1–5 dpf	–	Awake or 0.01% tricaine	–	DsRed-Express (microglia) GFP (neuron)	Confocal	20x	– 40–50 planes with 3 μm step	3–5 min –	Xu et al. (53)
Thy1-YFP^+/−^ mice (H-line) CX3CR1-EGFP^+/−^ mice	–	Thinned	Ketamine and xylazine	Cortex (~100 μm for neuron and ~200 μm for microglia)	YFP (neuron) EGFP (microglia)	Two-photon	60x (NA = 1.1)	512 × 512 pixel (66.7 × 66.7 μm) 0.75 μm step	– ~2 h	Yang et al. (46)
Thy1-YFP^+/−^ mice Thy1-GFP^+/−^ mice	1 mo	Thinned	Ketamine and xylazine	Motor cortex (100–200 μm)	YFP or GFP (neuron)	Two-photon	60x (NA = 1.1)	70 × 70 μm (512 × 512 pixels) 9 planes with 0.7 μm step	–	Yu et al. (47)

*mo, month-old; dpf, days post-fertilization; wo, week-old; P, post-natal day; –, not available*.

Since 2005, there has been increasing research on *in vivo* live imaging of microglia, and now thinned skull and open skull are recognized as the two major methods for preparing imaging window. The pros and cons of each method are well-summarized in the review article by Dorand et al. (48). Removing skull could stimulate microglia and change their state, and it requires several weeks for microglia to return to the physiological state (5, 16). The open skull method, on the other hand, has the advantage of a wider imaging depth and higher resolution than the thinned skull method (46, 49). Abnormal changes in microglial state induced by removing skull are critical not only because it affects the morphology and motility of microglia themselves, but also because cytokines released by microglia could affect the properties of surrounding cells. Although Isshiki and Okabe reported that they could not observe synaptic structures clearly using through the thinned skull (31), several studies have reported successful observation of synaptic structures such as dendritic spines *in vivo* through a thin-skull window (24, 44, 46, 47).

It is important to consider whether to use an open skull method or a thinned skull method, depending on the brain region or phenomenon being targeted. It should also be noted that both methods require technical proficiency of the experimenter to increase the success rate, and that there are difficulties in *in vivo* live imaging to achieve a high level of imaging between specimens.

#### Anesthesia

Proper anesthesia of animals is essential for *in vivo* live imaging. Live imaging with awake animals using head fixation is possible, and although many papers have been published on awake imaging, there is no doubt that anesthesia is useful for reducing motion artifacts, which may affect live imaging of microstructures such as synapses and microglial filopodia, and achieving more stable imaging. However, there is still the problem that the activity of each brain cell (including neuronal firing and microglial motility) is affected by anesthesia. For example, anesthesia suppresses neuronal firing activity (50) and alters the turnover rate of dendritic spines (40, 51). In recent years, it has been reported that anesthesia affects not only neuronal activity but also microglial motility, and the way in which this is affected depends on the type of anesthesia. For example, isoflurane, one of the most commonly used anesthetics, reduces the motility of microglial processes by inhibiting the potassium channel THIK-1 in acutely sectioned brain slices, whereas urethane has no such effect (52). On the other hand, live-imaging studies have also suggested that isoflurane enhanced microglial surveillance by increasing the length and velocity of surveilling processes as well as the frequency of extension and retraction of processes during both physiological and pathological conditions (33, 41, 42). It has also recently been shown that ketamine/xylazine, which is as frequently used as isoflurane, reduces the morphological complexity and process motility of microglia under physiological conditions (29). Morphological changes in microglia are likely to result in functional changes, and it is almost certain that anesthesia will influence experimental results and their interpretation. It is hoped that further research will reveal which anesthetics are able to preserve microglial properties in the awake state.

### Phenomena Verified by *in vivo* Live Imaging

#### Colonization of Microglia

During the embryonic period, microglia migrate from the yolk sac to the brain parenchyma. In 2016, Xu et al. observed the migration of microglia in zebrafish larvae using live imaging (53). The advantage of using zebrafish larvae is their small size and high transparency. This makes it possible to obtain high-resolution images of cell motility *in vivo*. Another attraction is the wide range of transgenic lines available, allowing live imaging of cells without viral infection or injection of fluorescent reagents. In addition, molecules involved in the detection and phagocytosis of dead cells by microglia are conserved in vertebrates, and if the observed phenomenon is carefully interpreted, it may be possible to extrapolate and validate findings acquired from studies using zebrafish to mammals. Xu et al. found that microglial progenitor cells infiltrate the optic tectum through a circulation-independent pathway. In addition, based on the fact that neuronal death occurs in the optic tectum during microglial migration, the authors investigated the possibility that apoptotic cells are involved in microglial migration. Suppression of neuronal cell death by overexpression of the anti-apoptotic protein bcl-2 significantly suppressed microglial infiltration into the optic tectum. Furthermore, ATP and lysophosphatidylcholine (LPC) released from apoptotic cells were identified as molecules that promote infiltration.

Casano et al. published a paper around the same time as Xu et al. in which they also examined the relationship between developmental microglial colonization and apoptotic cells (15). Suppression of neuronal cell death by administration of the caspase inhibitor Z-VAD-fmk or promotion of neuronal cell death by UV irradiation inhibited or promoted microglial infiltration to the brain parenchyma, respectively. Next, they showed that inhibition of nucleic acid release by pannexin-1a knockdown or inhibition of purinergic receptors by suramin suppressed microglial infiltration. A study using live imaging of acute slices of mouse embryonic cerebral cortex (54) reported that the migration rate of microglia is higher in the earlier embryonic period, which may be due to differential concentrations of apoptotic cell-derived molecules.

Contrary to the findings by Xu et al. (53), there are reports suggesting that the infiltration of microglia into the parenchyma is circulation-dependent in the fetal mouse brain (55). Furthermore, in live imaging of acute hippocampal slices prepared from 1-week-old mice, Eyo et al. showed that knockout of the apoptotic protein BAX did not affect microglial morphology or motility, and concluded that apoptotic cells do not regulate microglial dynamics (56). Thus, it is quite possible that the same phenomenon has different underlying mechanisms in different species.

Askew et al. examined the mechanism of microglial population maintenance by live imaging over 22 days (12). Interestingly, immediately after division, newborn microglia exhibited a 2-fold higher mortality rate than resident microglia. Furthermore, the authors found that microglial division is spatiotemporally coupled with microglial mortality. The molecular mechanism of this coupling has not been clarified, but CSF1R, which is essential for microglial survival, and IL-1R, which promotes microglial repopulation, have been considered as candidates.

Microglial proliferation is known to be enhanced in the brain following neurodegeneration and injury. Füger et al. developed an impressive method to examine the rate of microglial proliferation and turnover (22). The authors successfully fluorescently labeled single microglia within the field of view by using CD11b-CreERT2;R26-tdTomato mice with modified tamoxifen administration. They followed the same microglial fate with biweekly imaging for 6 months and with monthly imaging afterwards (starting at 4 months of age). It was reported that both the proliferation and disappearance rates of microglia were 13%. The median survival time was 15 months, which means that approximately half of all microglia in mice are present for life. The authors also crossed CD11b-CreERT2;R26-tdTomato mice with APP/PS1 mice and examined microglia around the Aβ plaques (between 4 and 6 months of age). The rate of proliferation and disappearance of microglia around Aβ plaques approximately doubled (20%).

#### Microglial Contact to Other Type of Cells

One of the most important things that cannot be revealed without live imaging is how microglia interact with other cell types. By observing this interaction, we can discover new phenomena and get ideas for examining cell-cell signaling. In a report by Nimmerjahn et al. (1), the authors provided quantitative data on the process dynamics of microglia and even mentioned their interactions with other cell types. First, the authors labeled astrocytes, one of the major glial cells, with the red fluorescent dye sulforhodamine 101 (SR101) and showed that astrocytes, in contrast to microglia, showed virtually no change in morphology of their soma and primary processes. However, it should be noted that SR101 does not label the fine, distal astrocytic processes which are motile and only the proximal branches are labeled. They also captured the microglia coming into contact with the area where the SR101 signal was missing, presumably the cell bodies of the neurons, and with the area surrounding the astrocytes, presumably the blood vessels, which are surrounded by processes of the astrocytes. When the blood-brain barrier was disrupted by laser irradiation, microglial processes accumulated in the irradiated area, suggesting that microglia may also interact with blood vessels.

The improved imaging resolution has enabled real-time observation of microstructures such as microglial filopodia and synapses. Wake et al. performed *in vivo* two-photon imaging of the sensory or visual cortex in mice (3). They found that microglia make contact with axonal boutons and dendritic spines. It was shown that microglial contact with synapses increased with elevated neural activity by manipulating neural activity through visual stimulation and thermoregulation in mice. Furthermore, the duration of microglial contact with boutons was prolonged after the induction of ischemia, and more than half of the contacted boutons were lost. These results indicate that microglia may monitor synapses by contact and remove abnormal synapses. These results suggest that the ramified form of microglia is suitable for monitoring synapses.

Tremblay et al. also focused on the synaptic monitoring by microglia and performed *in vivo* two-photon imaging of microglia and dendrites in the visual cortex (44). When mice were transferred from dark to light environments to increase neural activity, microglial motility increased. Electron microscopy revealed that microglial contact with synapses was increased in mice transferred from dark to light environments. Furthermore, live imaging confirmed that the size of the spine increased during microglial contact and decreased after contact. Spines with microglial contact had a higher rate of subsequent disappearance than spines without contact, suggesting that microglia are responsible for synaptic removal. The involvement of microglia in synapse elimination predicted from the work of Wake et al. (3) and Tremblay et al. (44) using *in vivo* live imaging was later proved histochemically by the work of Paolicelli et al. (57) and Schafer et al. (58) who found phagocytic inclusions containing synaptic elements in microglia.

It had also been suggested that microglia modulate synapse formation through the release of BDNF (37), but it remained unclear whether microglial contact with synapses is important for synapse formation. To test that point, Miyamoto et al. performed *in vivo* multiphoton imaging of layers II and III of the sensory cortex of post-natal (P) 8 to 10-day-old mice, where synaptogenesis is active (34). The formation rate of filopodia was significantly higher in dendritic shafts contacting microglia than in areas without microglia. On the other hand, administration of minocycline, a derivative tetracycline that inhibits pro-inflammatory response of microglia, did not change the frequency of microglial contact with the dendrites but decreased the probability of filopodia formation. The filopodia formation rate was higher when the microglial contact with dendrites was accompanied by a local increase in Ca^2+^ concentration on the dendrites. Furthermore, removal of microglia by the Dox system during P5-11 reduced the number of functional synapses, indicating the need for microglia in synapse formation. The authors suggested that microglial state is important for the increase in Ca^2+^ concentration and subsequent spine formation in dendrites on contact because minocycline reduced the expression of the Ca^2+^-binding protein Iba1 mRNA in microglia.

Together, *in vivo* live imaging studies of microglia-synapse interactions have provided direct evidence that microglia regulate not only synapse removal, but also synapse formation.

Stowell et al. examined the effect of one of the major neurotransmitters, norepinephrine, on microglia-synapse interaction (41). Pharmacological experiments showed that norepinephrine caused microglial process retraction and inhibition of bulbous tip formation by activating β2-adrenoceptors expressed in microglia. Furthermore, activation of β2-adrenoceptors inhibited ocular dominance in the visual cortex due to monocular shielding and reduced the number of microglial contacts with dendritic spines. These results indicate that β2-adrenergic receptor-dependent microglial contact with the spine may modulate synaptic plasticity.

There are numerous reports of microglia interacting with neurons in response to neural activity, and live imaging studies have been useful in targeting this relationship. Performing *in vivo* imaging of zebrafish, Li et al. presented an important finding that microglia can both monitor and manipulate neural activity (32). When glutamate uncaging increased the neural activity, microglial processes were attracted toward the active neurons. In doing so, bulbous tips were formed at the tips of microglial processes. Furthermore, in combination with Ca^2+^ imaging, it was shown that neural activity was reduced following microglial contact. Visualization of microglial Rac using the FRET system further revealed that Rac activity was increased at the microglial process tip as neural activity increased, indicating that this is required for microglial contact to neurons and the formation of a bulbous tip.

Badimon et al. showed that microglia reduce neural activity via the release of adenosine (13). Live imaging of microglia, neuronal axons, and bouton-like structures revealed that increased neural activity via the DREADD system promotes microglial process extension via activation of P2Y12.

#### Phagocytosis

Since the persistence of dead cells in the brain parenchyma threatens the homeostasis of brain functions, it is important to elucidate the mechanism of dead cell removal by microglia. Peri et al. performed *in vivo* live imaging of zebrafish larvae and observed microglial motility (38) to examine the involvement of microglia in the removal of dead cells. Animals knocked out for v0-ATPase a1, which encodes a component of the vacuolar ATPase (V-ATPase), a multisubunit enzyme that mediates the acidification of eukaryotic intracellular organelles, showed reduced digestion of dead cells incorporated into microglia. In addition, phagosomes incorporating dead cells fused with each other during the digestion process. Furthermore, knocking out v0-ATPase a1 also inhibited the fusion of phagosomes and lysosomes, i.e., the formation of phagolysosomes. This study is impactful in that it unveils the process of digestion of dead cells by microglia and shows that microglia have the ability to not only take in dead cells but also digest them.

Morsch et al. conducted live imaging of spinal cord of zebrafish and showed microglial migration toward and uptake of irradiated neurons upon induction of neuronal cell death by UV irradiation (35). In combination with AnnexinV-mVenus, it was revealed that irradiated neurons underwent apoptosis after being taken up by microglia. Furthermore, the morphology of the microglia changed to an amoeboid shape during neuronal phagocytosis and returned to a stellate morphology after the digestion of neurons. The authors also compared other parameters, such as migration speed and microglial size.

Traumatic brain injury (TBI) also causes pronounced neuronal cell death. Microglia remove dead cells through phagocytosis, but it is not clear whether this phenomenon is protective or damaging to the brain. Herzog et al. performed live imaging of juvenile zebrafish and confirmed that microglia migrate to the injury site within minutes after TBI and phagocytose dead cells (27). In addition, the KO of P2Y12, which is necessary for microglial migration to the injury site, and inhibition of the phosphatidylserine (PS) receptor BAI1 reduced microglial phagocytosis of dead cells and accelerated subsequent secondary cell death in the injury site. These results suggest that the removal of dead cells by microglia is neuroprotective.

Damisah et al. observed the response of microglia and astrocytes to dead neurons in live imaging using photochemical techniques to induce apoptosis in single cell (17). Microglia and astrocytes were observed in separate mice; while microglia phagocytosed cell bodies and proximal dendrites, astrocytes phagocytosed distal dendrites and debris. The authors showed that knocking out the receptor tyrosine kinase Mertk delayed the recognition of dead cells by microglia. In microglia-removed mice, multiple astrocytes around dead cells cooperated in the phagocytosis of cell bodies. This suggests that astrocytes function in a supportive manner for microglia in the phagocytosis of dead cells. Further simultaneous live imaging of microglia and astrocytes is needed to understand the interaction of the two major phagocytes in the brain.

Abiega et al. examined the effect of neuronal hyperexcitability on the clearance of apoptotic cells by microglia (11). Twenty-four hours after KA administration to the hippocampus, process motility (velocity) of cortical microglia decreased. In addition, using immunostaining, FACS and live imaging of acute slices, the authors found that ATP released from hyperexcitable neurons masked the eat-me signal from apoptotic cells, which inhibited phagocytosis of apoptotic cells by microglia. Furthermore, the delayed clearance of apoptotic cells exacerbated the inflammatory response, suggesting that microglial clearance of apoptotic cells is important for maintaining brain homeostasis in the epileptic brain. However, even though neuronal death and microglial phagocytosis of dead cells were prominent in the dentate gyrus and hippocampus, the *in vivo* live imaging in this study was performed in the cortex, not the hippocampus, highlighting the technical difficulty of imaging deep brain regions.

Grier et al. investigated the possibility that neural activity mediates phagocytosis by microglia in the mammalian olfactory bulb where the lifelong activity-dependent plasticity occurs (23). Dopaminergic neurons in the glomerular layer are particularly plastic, with a sharp decrease in tyrosine hydroxylase expression levels and dopamine production, followed by a decrease in the number of dopaminergic neurons upon blockade of sensory input (59, 60). The authors performed *in vivo* live imaging and found that the blockade of sensory input after nasal obstruction resulted in morphological change of microglia (increased perimeter length) and an increased change rate of perimeter length (23). These results suggest that the reduced neural activity caused by the blockade of sensory input increases the frequency of microglial process extension and retraction. Although not through live imaging, in the nasal obstruction group, the microglia appeared to cover the cell bodies of the dopaminergic neurons, some of which were completely incorporated into the microglia. It was also shown that the synapses of dopaminergic neurons were incorporated into the microglia. These results indicate that the activity-dependent plasticity of the olfactory bulb may be regulated by microglia.

Myelin, which covers axons and controls the velocity of nerve conduction, is also a highly plastic structure that should be studied to further elucidate the interaction between axons and microglia. Although there have been few reports of *in vivo* live imaging of myelin at present, Hill et al. successfully observed stable myelin in label-free, *in vivo* live imaging in genetically engineered mice with fluorescently labeled myelin (28). The authors found that the production of oligodendrocytes and the structural plasticity of myelin occur throughout life. They also showed that cell death of oligodendrocytes and myelin degradation occur in old mice. Furthermore, live imaging of mice in which myelin, oligodendrocytes, and microglia were fluorescently labeled revealed that myelin debris produced in old mice is removed by microglia via phagocytosis.

Recently, it has been suggested that microglia also phagocytose tumor cells. Glioblastoma multiforme is a malignant brain tumor and is considered incurable. Tumor-associated macrophages and microglia (TAMs) are major cell types in tumorigenesis and have been shown to promote tumor growth. Therefore, it has been hypothesized that regulating TAM function might conversely inhibit tumor growth. Hutter et al. previously suggested that suppressing SIRPα-CD47 signaling, a “don't eat me” signal, and promoting tumor phagocytosis by TAM could treat a variety of tumors (61). However, this antitumor effect has been thought to be largely due to macrophages infiltrating from the periphery, and the contribution of brain-resident microglia has not been clarified. In 2019, Hutter et al. attempted to clarify this point by using mice that can distinguish between microglia and macrophages (30). The authors found that CD47 inhibition also promoted tumor phagocytosis by microglia, and *in vivo* live imaging showed that CD47 inhibition reduced the number of microglial processes, increased process straightness, and reduced the speed of process extension and retraction. These changes in microglial morphology and motility indicated increased microglial contact with tumor cells.

#### Second Messenger Signaling in Microglia

The ability to observe second messenger signaling within microglia in real time would reveal what molecules are involved in microglial surveillance and directed movement. Such studies would be a first step toward modulating brain function through microglial regulation. Intracellular Ca^2+^ dynamics in microglia had been difficult to observe because of the difficulty in efficiently introducing Ca^2+^ indicator and adeno-associated viruses into microglia, but Eichhoff et al. succeeded in visualizing microglial Ca^2+^ dynamics for the first time (21). The authors loaded microglia with a calcium indicator, Oregon green BAPTA 1 (OGB-1). In microglia, almost no spontaneous Ca^2+^ fluctuations or Ca^2+^ response to increased activity of adjacent neurons, which was induced by bicuculline, was observed. On the other hand, physical damage by electrodes to neurons increased the Ca^2+^ concentration in nearby microglia. It was also suggested that the increase in Ca^2+^ in microglia was due to the activation of the ATP receptor P2Y. Furthermore, the latency from neuronal damage to Ca^2+^ elevation was significantly shorter in microglia than that in astrocytes, suggesting that fluctuations in intracellular Ca^2+^ concentration underlie the agile response of microglia to tissue damage.

Pozner et al. monitored the intracellular Ca^2+^ concentration by GCaMP5G while observing microglial morphology with tdTomato using the PC::G5-tdT mouse line (39). Spontaneous increases in Ca^2+^ concentrations were almost non-existent, but the frequency of elevated Ca^2+^ concentrations increased ~8-fold when the microglia were stimulated by LPS. This increase in Ca^2+^ concentration was observed simultaneously in several microglia. Furthermore, the rates of process extension and retraction of microglia with elevated Ca^2+^ concentrations were reduced compared to those of microglia without Ca^2+^ concentration fluctuations.

As mentioned earlier, observing intracellular Ca^2+^ concentrations in microglia has been technically challenging. Brawek et al. successfully visualized microglial Ca^2+^ concentrations (14) using a different method than Eichhoff et al. (21) and Pozner et al. (39). Brawek et al. took advantage of the lack of microRNA-9 expression in microglia to create and apply a lentivirus that expresses the Ca^2+^-indicator protein Twitch-2B in a microRNA-9-dependent manner. They found that the *in vivo* quiescent microglia showed stable and low Ca^2+^ concentrations. Since similar results were obtained in the study by Pozner et al. using different tools, it is likely that there is almost no Ca^2+^ fluctuation in microglia (at least under physiological conditions). In addition, Ca^2+^ concentrations were elevated in acute slices and dispersed cultures immediately after excision from the brain. This was accompanied by increased expression levels of CD68 and IL-1β, suggesting that intracellular Ca^2+^ concentration is an indicator of microglial proinflammatory response. The authors also confirmed that *in vivo* laser-induced tissue damage and ATP injection increased microglial Ca^2+^ concentrations in microglia, suggesting an association between directed process extension to injured areas and intracellular Ca^2+^ concentrations.

Olmedillas Del Moral et al. applied single-cell electroporation of the Ca^2+^ indicator Oregon Green 488 to microglia and observed intracellular Ca^2+^ concentrations in microglia (36). In 2 to 4-month-old (“young adult”), 9 to 11-month-old (“middle-aged”), and 18 to 21-month-old (“old”) mice, the frequency and amplitude of spontaneous Ca^2+^ concentration increases were found to be highest in middle-aged adults. In addition, the speed of process extension toward ATP increased the most in the old group, but the correlation between distance from the ATP injection site and the process speed was lowest in the old group, indicating that the ATP responsiveness varied. These results indicate that the effect of aging may be different between individual microglia.

Umpierre et al. observed Ca^2+^ dynamics in microglia of awake mice in 2020 (45). The authors achieved microglia-specific expression of GCaMP using CX3CR1-CreER mice. Awake mice also showed no spontaneous increase in Ca^2+^ concentration. Interestingly, an increase in Ca^2+^ with process elongation was confirmed in response to both an increased and decreased neuronal activity. Although the dynamics of Ca^2+^ in microglia has been observed by various methods, no study has yet addressed the molecular mechanism that control Ca^2+^ dynamics in microglia.

#### Response of Microglia to Damage

Brain injury is often accompanied by inflammation. As immune cells in the brain, microglia may both exacerbate and suppress inflammation. *In vivo* imaging of microglial dynamics in and around injured sites will highlight the study of microglia as a potential therapeutic target for brain injury and the subsequent brain diseases. Davalos et al. is the first report that succeeded in capturing live ramified microglia *in vivo* (2). In addition to this observation, the authors examined the changes in microglial dynamics caused by tissue damage using laser irradiation and glass electrodes. Furthermore, through a combination of pharmacological treatments, it was shown that ATP released from the site of injury causes microglial process extension via activation of purinergic G protein-coupled receptors in microglia.

Haynes et al. examined the molecular mechanism of directed movement of microglial processes to the site of injury (26). Using KO mice, they demonstrated that P2Y12, which is unique in that it is a Gi-type receptor among mostly Gq-type P2Y receptors, is required for process extension toward the site of injury. Interestingly, knocking out P2Y12 did not affect the basic surveillance by microglia. Therefore, this study is significant in that it shows that directed process extension to the injury site and surveillance may be regulated by different molecular mechanisms. In the same paper, the authors also performed live imaging of acute slices and showed that P2Y12 expression levels were reduced in correlation with microglial state change.

In the white mater of the spinal cord, the induction of microglial processes at the site of injury was also tested (20). The results showed that elevated ATP and NO levels at the site of injury promoted the extension of microglial processes. Stable live imaging of spinal cord was considered to be difficult due to motion artifacts; Davalos et al. established a spinal cord live imaging system that overcame the above problems by increasing the depth of anesthesia and improving the fixation device (18). However, it should be noted that ketamine and xylazine used as anesthesia could affect the motility of microglia.

Davalos et al. investigated the functional changes in microglia that occur in the diseased brain by *in vivo* live imaging (19). The authors examined the causes of blood-brain barrier (BBB) damage, microglial state change and neurodegeneration in multiple sclerotic brains and the mechanisms by which they cause axonal damage. In addition, *in vivo* live imaging revealed that microglial accumulation and myelin shedding occurred before the manifestation of paralysis, a symptom of multiple sclerosis. Furthermore, inhibition of fibrinogen formation by the anticoagulant lepirudin, genetic removal of fibrinogen, and removal of the binding site of fibrinogen to CD11b/CD18 (Fib–/–, Fibγ390-396A mice) inhibited the perivascular accumulation of microglia and axonal damage. This study reaffirms the importance of live imaging because this technique unveiled the temporal relationship between microglial accumulation and state change, both of which are causes of multiple sclerosis.

Taylor et al. examined the effect of type I diabetes on the repair of damaged blood vessels by microglia (43). In a mouse model of diabetes, laser irradiation did not cause migration of microglia to the damaged vessels. And the loss of vascular repair by microglia exacerbated secondary vascular leakage, suggesting that microglia are important for the suppression of vascular damage. Furthermore, they found that an increase in IFN-γ levels in the blood of a mouse model of obese reduced the expression level of P2Y12 in microglia and suppressed the migration of microglia to damaged blood vessels. Since live imaging of intact blood vessels can only be performed *in vivo, in vivo* live imaging may be the most suitable method to verify the relationship between vascular injury and microglia.

### Achievement and Limitation of *in vivo* Live Imaging

In summary, *in vivo* imaging studies have revealed the dynamics of ramified microglia and contributed to our understanding of the dynamics and significance of microglial motility. For example, it was revealed that microglia could migrate long distance and intracellular Ca^2+^ activity of microglia was clarified. Also, it was shown that microglia change their dynamics in response to various transmitters, neural activity, injury, and interaction between other cell types.

It is expected that advances in genetic modification and imaging technology will further refine *in vivo* live imaging methods for observing intact microglial dynamics, but various technical limitations should not be overlooked at this point. First, most of the live imaging in the studies described in section *In vivo* Live Imaging (see Table 1 for Experimental Conditions) was performed in the vicinity of the brain surface, which highlights the difficulty of deep brain imaging. The cortical area is a good place to test the relationship between neural activity and microglial dynamics, as well as the relationship between animal behavior and microglial dynamics, since neural activity can be altered by the introduction of sensory stimuli such as visual and auditory stimuli. Live imaging of microglia in deeper brain regions such as the hippocampus and amygdala, which control learning, memory, and emotions, may lead to the discovery of region-specificity of microglial motility and new significance of microglial surveillance in brain functions. Gu et al. showed that *in vivo* live imaging of the hippocampus is possible by aspiration of the cortex (25). Microglia in the hippocampus have been shown by immunostaining to exhibit morphology such as reduced process length and hypertrophy of the cell body after surgery, but to return to the surveillant state after 10 days. Badimon et al. performed *in vivo* neuronal calcium imaging in the dorsal striatum using the gradient-index (GRIN) lens (13). Thus, it is expected that live imaging studies of microglia in deep brain regions will be further advanced by using techniques such as the GRIN optics and cortical aspiration, and that details of microglial dynamics related to brain structure and function will be revealed.

Furthermore, it seems difficult to combine *in vivo* live imaging with the cell function manipulation by pharmacological and optogenetic methods, because the presence of bones in the thinned skull or the presence of glass coverslips in the open skull will limit what can be done from the brain surface.

**Figure 2:**
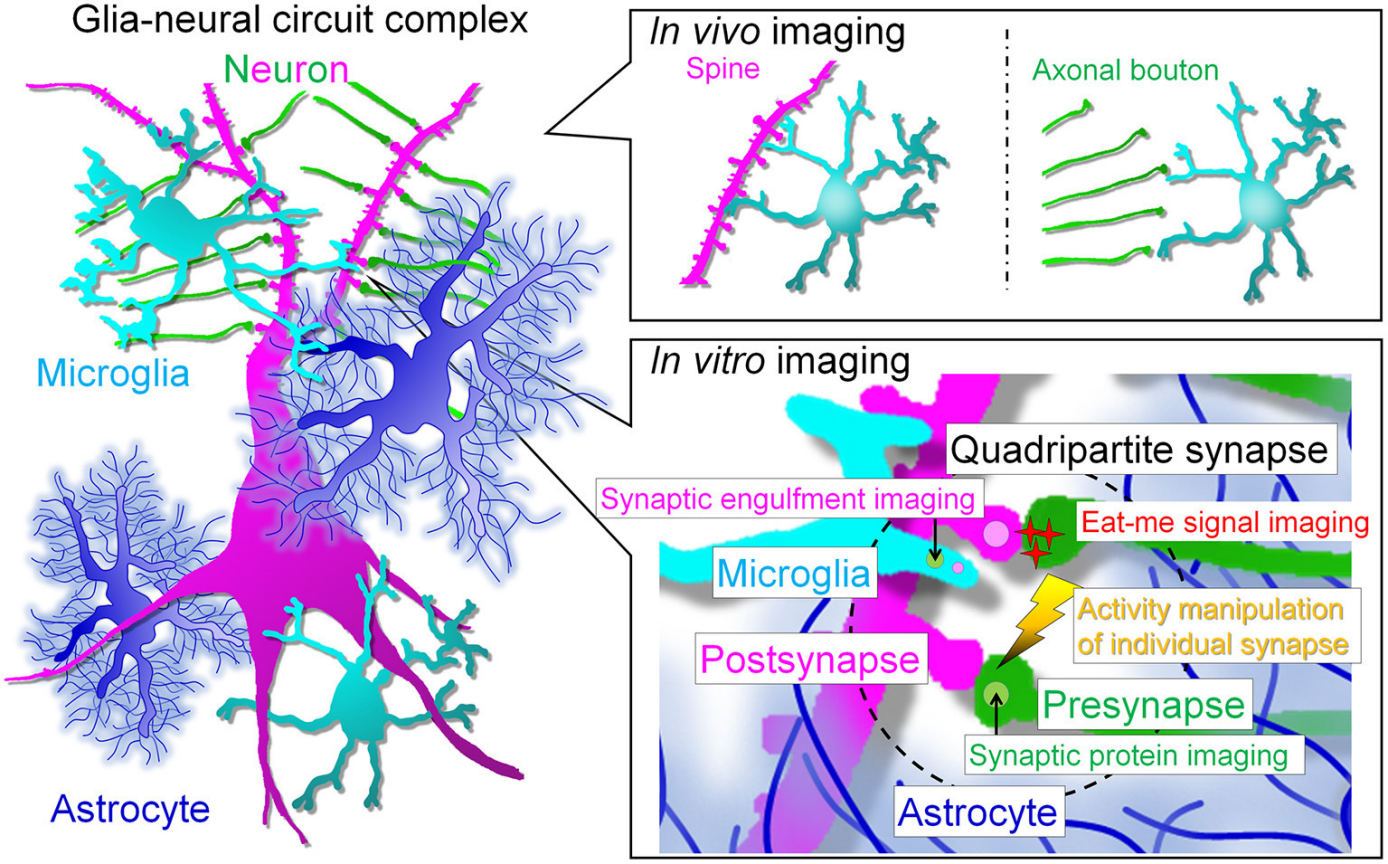
Prospects for live imaging of glial cell-neural circuit complexes. *In vivo* multiphoton imaging studies have revealed that microglia interact with spines and axonal boutons. *In vivo* live imaging has greatly advanced the field by revealing the correlation between interactions and neural activity and has enhanced research interest in microglia. However, the next step will be to assess the interaction of each cell type at the synaptic site and the molecular mechanism of the interaction. For this purpose, we need to perform simultaneous high-resolution imaging of the quadripartite synapse, which consists of microglia, astrocytes and pre- and post-synapses. The simultaneous imaging of synapses, neuronal membranes, and microglia will help to answer the remaining major question of whether neurites are snipped off during synaptic phagocytosis. In addition, to understand the molecular mechanism of synaptic competition, it is necessary to control activity at the level of individual synapses. *In vitro* systems will facilitate live imaging with multiple colors, high resolution, and local stimulation, but a major barrier must be crossed: the establishment of glial cell culture systems that maintain *in vivo* morphology and gene expression. The realization of such systems is eagerly awaited to clarify the relationship between glia and neurons, especially synapses.

Problems of *in vivo* imaging such as limited observation regions, low resolution, and difficulty in stimulation have been improved. Thus, complementary understanding of the data obtained by *in vivo* imaging and *in vitro* imaging, which has advantages such as observation at super-resolution level and manipulation at high spatiotemporal resolution, will help us to understand the mechanism underlying microglia-neuron interaction such as synapse elimination (Figure 2).

## *Ex vivo* Live Imaging

Live imaging of microglia *ex vivo* has been attempted as a way to overcome technical difficulties *in vivo*. In section *Ex vivo* Live Imaging, we will present studies using live imaging of brain slices. Perhaps the most significant advantage of slice imaging is that the structures in the brain (cell layers and projection pathways) are preserved. This is important for validating the direction of microglial migration and helps facilitate the extrapolation of results obtained with slice imaging to *in vivo*. In the following sections, we introduce acute slice imaging, in which sections cut out from the brain are observed immediately after preparation, and cultured slice imaging, in which sections are cultured and observed at any given time.

### Acute Slice

#### Alterations of Microglial Process Dynamics by ATP and Neurotransmitters

Wu et al. examined the molecular mechanisms regulating microglial process motility by the ATP-P2Y receptor signaling pathway (76) (Table 2). Simultaneously, observing microglial motility by confocal microscopy and performing whole-cell patch-clamp recordings of microglia, the authors found that process extension to ATP correlated with the outward potassium current associated with P2Y receptor activation. It was also shown that both P2Y receptor activation and outward potassium currents are required for process extension to ATP and basal surveillance. Furthermore, it was shown that the PI3K signaling pathway is important for ATP-induced chemotaxis. Swiatkowski et al. showed that the regulation of process extension by outward potassium currents functions similarly in the microglial response to neuronal damage (75).

**Table 2:** Experimental conditions of live imaging of microglia *ex vivo* (acute slice).

**Animal**	**Age**	**Anesthesia**	**Region**	**Thickness ** **Depth from the surface**	**Cellular visualization**	**Microscopy**	**Objective lens**	**Resolution (x, y) ** **z stack**	**Interval time ** **Total time**	**References**
CX3CR1-GFP^+/−^ mice	P30-40	–	Hippocampus	350 μm –	GFP (microglia)	Two-photon	40x (NA = 1.0)	– 11–25 μm thick	25 or 60 s ~1 h	Avignone et al. (62)
CX3CR1-GFP^+/−^ mice	P45-180	Isoflurane	Hippocampus	300 μm 150 ± 25 μm	GFP (microglia)	Two-photon	20x (NA = 1.0)	512 × 512 or 1,024 × 1,024 pixel 1–2 μm step	–	Bernier et al. (9)
C57BL/6 mice	6–8 wo	–	Coronal section	300 μm –	Alexa 488, 568 isolectin B4 (microglia)	Confocal	20x 40x	175 × 175 μm or 350 × 350 μm 40 μm	1.5 min –	Carbonell et al. (63)
CX3CR1-GFP^+/−^ mice	P40-120	Halothane	Hippocampus	300 μm 150 ± 25 μm	GFP (microglia)	Two-photon	40x (NA = 1.0)	512 × 512 pixel 15 planes with 2 μm step	1 min –	Dissing-Olesen et al. (64)
CX3CR1-GFP^+/−^ mice	P5-7	–	Hippocampus	400 μm 45–60 μm	GFP (microglia)	Confocal and multi-photon	20x (NA = 0.7)	775 × 775 μm 15 planes with 3 μm step	3–10 min –	Eyo et al. (65)
CX3CR1-GFP^+/−^;Thy1-YFP^+/−^ mice	4 wo	–	Hippocampus	300 μm 50–100 μm	GFP (microglia) YFP (neuron)	Two-photon	40x (NA = 0.8)	–	–	Eyo et al. (66)
CX3CR1-GFP^+/−^ mice	P2-6	–	Hippocampus	400 μm 45–60 μm	GFP (microglia)	Confocal	20x (NA = 0.7)	775 × 775 μm 15 planes with 3 μm step	10 min –	Eyo et al. (56)
CX3CR1-GFP^+/−^;Thy1-YFP^+/−^ mice	3–5 wo	–	Cortex	300 μm 50–120 μm	GFP (microglia) YFP (neuron)	Two-photon	40x (NA = 0.8)	– 15 planes with 3 μm step or 10 planes with 2 μm step	30 s –	Eyo et al. (67)
CX3CR1-GFP^+/−^ mice	3–8 mo	–	Retina	–	GFP (microglia)	Confocal	40x (NA = 0.8)	512 × 512 pixel –	10 s –	Fontainhas et al. (68)
CX3CR1-GFP^+/−^ mice	1–4 mo	–	Cortex	200 μm	GFP (microglia)	Confocal	60x	– 30–50 planes with 1 μm step	1 min –	Gyoneva et al. (69)
CX3CR1-GFP^+/−^ mice	–	–	Substantia nigra	200 μm	GFP (microglia)	Confocal	20x (NA = 0.50)	– 31 planes with 1 μm step	30–60 s 20 min	Gyoneva et al. (70)
SD rats Iba1-GFP^+/−^ mice	P12 P15-27	–	Hippocampus	300 μm ~50–100 μm	Isolectin B4-Alexa 594 or GFP (microglia)	Two-photon	20x (NA = 1.0)	512 × 512 pixel (0.49–0.39 μm/pixel) 21–31 planes with 2 μm step	60 s	Madry et al. (52)
MacGreen/cd39^−/−^ mice	–	–	Somatosensory cortex	300 μm –	MacGreen (microglia)	Two-photon	40x (NA = 0.8)	307 × 307 μm 21 planes with 3 μm step		Matyash et al. (71)
SD rats Iba1-GFP^+/−^ mice	P12 P15-27	–	Hippocampus	300 μm ~50–100 μm	Isolectin B4-Alexa 594 or GFP (microglia)	Two-photon	20x (NA = 1.0)	512 × 512 pixel (0.49–0.39 μm/pixel) 21–31 planes with 2 μm step	60 s	Zhao et al. (72)
CX3CR1-GFP^+/−^;Thy1-YFP^+/−^ mice	P28-40	Isoflurane	Hippocampus	–	GFP (microglia) YFP (neuron)	Confocal	40x (NA = 1.0)	512 × 512 pixel (0.39 μm/pixel) 11 planes with 1 μm step	30 s 80 min	Pfeiffer et al. (73)
CX3CR1-GFP^+/−^ mice	3 mo~	Euthasol	Spinal cord	350 μm –	GFP (microglia) Alexa Fluor 555 (neuron)	Two-photon	25x (NA = 0.95)	512 × 512 pixel 31 planes with 1.5 μm step	30–60 s 30–60 min	Rotterman and Alvarez (74)
Rats	P3-14	–	Hippocampus	400 μm –	FITC-IB 4 FITC-IB 4 FITC-IB 4 FITC-IB_4_ (microglia_)_	Confocal	63x (NA = 1.2)	512 × 512 or 1,024 × 1,024 pixel 8–18 planes (~60 μm)	2–5 min 30–90 min	Stence et al. (6)
CX3CR1-GFP^+/−^ mice	3–6 wo	–	Coronal section	300 μm 50–100 μm	GFP (microglia)	Two-photon	40x (NA = 0.8)	1,024 × 1,024 pixel (0.16 μm/pixel) 15 planes with 3 μm step	1 min –	Swiatkowski et al. (75)
CX3CR1-GFP^+/−^ mice	E12.5, 14.5, 17.5	–	Coronal section	300 μm 50 μm	GFP (microglia)	Confocal	20x (NA = 0.5)	1,024 × 1,024 pixel 72 μm with 8 μm step	2 min 1 h	Swinnen et al. (54)
CX3CR1-GFP^+/−^ mice	8–10 wo	Halothane	Cortex	300 μm –	GFP (microglia)	Confocal	40x (NA = 0.8)	– 8–10 planes with 2 μm step	1 min –	Wu et al. (76)
rd10;CX3CR1-GFP^+/−^ mice	P21-24	–	Eyecup	–	GFP (microglia) PI (dead cell) Hoechst (nuclei)	Confocal	40x or 60x	1,024 × 1,024 pixel –	1 min 2 h	Zhao et al. (77)

*wo, week-old; mo, month-old; P, post-natal day; –, not available*.

Molecules that act in the opposite way to ATP have also been studied. Gyoneva et al. tested the effect of norepinephrine on the motility of microglial processes: bath application of ATP induced microglial process elongation, whereas norepinephrine treatment retracted them (69). Microglia extend their processes to the site of injury in an ATP-P2Y12 signaling-dependent manner. In 2014, Gyoneva et al. tested this by preparing acute slices of mice that had been subjected to inflammatory conditions and performing live imaging (70). In the control group, microglial processes extended to the injured site, but this phenomenon was inhibited when inflammation was induced by prior LPS administration. In addition, MPTP treatment, which induced dopaminergic neuronal death and microglial state change, also inhibited process extension to the injured site. Furthermore, LPS and MPTP treatment increased adenosine A2A receptor expression in microglia and suppressed process extension to the injured site. Because adenosine retracts microglial processes via A2A receptors, treatment with preladenant, an antagonist of the A2A receptor, along with MPTP treatment, rescued the inhibition of process extension to the injury site.

Dissing-Olesen et al. showed that ATP released from neurons upon NMDAR activation attracts microglial processes (64). Pharmacological experiments showed that the induction of the microglial process was independent of ATP and NO release from Pannexin1 and astrocytic connexins associated with NMDAR activation. The formation of a bulbous tip-like structure at the tip of the microglial process upon ATP treatment indicates that the microglial processes may contact activated neurons via the bulbous tips, and *in vivo* live imaging showed similar results. Eyo et al. showed that microglial process induction was also enhanced when neural activity was enhanced by kainic acid (66). Furthermore, they found that inhibiting microglial process number increases and process attraction by P2Y12 KO worsened kainic acid-induced seizures. These results suggest that microglial-neuronal contact in the epileptic brain has a neuroprotective effect.

Pfeiffer et al. examined the effect of increased neural activity associated with long-term potentiation (LTP) induction on the dynamics of microglia. After LTP induction, the number of microglial process branches increased, but the speed of extension and retraction was unchanged (73). Furthermore, live imaging of microglia and dendritic spines revealed an increase in the duration of microglial contact with spines after LTP induction, resulting in a decrease in the number of contacts. Furthermore, these changes were abolished by treatment with NMDA receptor antagonists. Taken together, these findings and the report by Dissing-Olesen et al. (64) suggest that neuron-derived ATP released in an NMDA receptor-dependent manner may regulate microglial-synaptic interactions.

While many studies have focused on microglial responses to excitatory neuron-derived neurotransmitters, Fontainhas et al. also focused on the inhibitory neurotransmitter GABA (68). Bath application of bicuculline, an inhibitor of ionic GABAA receptors, increased the length, extension and retraction rate and number of microglial processes and branches. On the other hand, a bath application of GABA reduced all these parameters. These results indicated that GABA decreases the motility of microglia.

#### Exploring the Regulatory Molecules of Microglial Dynamics Using Brain Injury Models

Zhao et al. tested the involvement of microglia in rod photoreceptor cell death in retinal degeneration (77). Live imaging of retinal explants in the rd10 mouse model of retinal degeneration showed that microglia phagocytose propidium iodide (PI)-positive and PI-negative rod photoreceptor cells. Microglial morphology and the way of phagocytosing rod photoreceptor cell varied, with amoeboid microglia forming a phagocytic cup near the cell body, and microglia with processes forming a phagocytic cup at the tip of the process or using lamellipodia.

Avignone et al. used a mouse model of kainic acid-induced status epilepticus and performed live imaging of microglia in hippocampal acute slices (62). Forty-eight hours after status epilepticus, microglia showed morphological changes such as enlarged soma and shorter process, but there was no change in surveillance or the extension speed of microglial processes to the site of injury. However, there was an increase in the extent to which a single process was explored and an increase in the speed of process extension to 2 Me-ADP. Eyo et al. also utilized a seizure model to examine the molecular mechanisms that regulate microglial contact with neurons and their relationship (67). Microglial process convergence (MPC), which is seen when microglia contact neurons, was used as an indicator of contact events. MPCs were increased when seizures were induced by kainic acid and pilocarpine. In addition, knocking out the CX3CR1 receptor, a fractalkine receptor, decreased MPCs, while treatment with its ligand CX3CL1 increased MPCs, indicating that the CX3CL1-CX3CR1 signaling pathway regulates microglial-neuron interactions. In addition, activation of CX3CR1 promoted IL-1β release from microglia. Furthermore, IL-1β promoted ATP release associated with increased neural activity and microglial process elongation via P2Y12 receptors. The aggravation of kainic acid- or pilocarpine-induced seizures in CX3CR1-KO mice suggests that microglia inhibit excessive neural activity by contacting neurons with elevated activity.

Carbonell et al. examined the response of mature microglia to thalamic damage (63). Acute slices were prepared 2, 3, 4, 6, and 8 days after the stab lesion and the thalamus was observed. The mobility of microglia around the injured site was highest after 3 days, but the direction of migration was random. As intracellular signaling, the authors focused on cysteine-cysteine (CC) chemokines involved in homing to the site of injury by leukocytes. They also showed that inhibition of CCR5 reduced migration speed and distance of microglia.

Eyo et al. focused on perinatal stroke and examined the effects of stroke-related deficiencies of oxygen and glucose on microglial motility (65). To model stroke, they treated acute hippocampal slices with hypoxia or oxygen-glucose deprivation (OGD). Both treatments reduced microglial motility and caused microglial death. Next, they compared the dynamics of microglia after OGD in slices of P2-3 and P6-7 and found that the former showed a lower cell death rate, a longer latency to death, and higher motility of microglia. These results suggest that the sensitivity of microglia to OGD increases with development.

#### Exploring the Molecules That Control the Morphology of Microglial Processes

The acute slice experimental system, which allows for more detailed pharmacological and genetic interventions than *in vivo* systems, including gene transfer, may contribute to the elucidation of mechanisms and molecules that could not be discovered *in vivo*. Matyash et al. found that knocking out of CD39 and CD73, which mediate the degradation of extracellular ATP to adenosine, reduced the length of microglial processes as well as the branching number (71). In acute slices of CD39-KO mice, the extension of microglial processes to ATP and the site of injury was suppressed. Microglial ramification was rescued by inhibition of the adenosine transporter and increased extracellular adenosine. A similar trend was observed in primary cultures of microglia. These results suggest that adenosine is important for the maintenance of the ramified morphology of microglia.

Madry et al. tested the possibility that the two-pore domain channel (THIK-1) regulates microglial process motility (52). THIK-1 is constitutively activated and is further activated upon activation of the P2Y12 receptor. Inhibition of THIK-1 activation reduced the ramification and surveillance of microglia but did not affect the extension of processes to ATP. In contrast, inhibition of P2Y12 receptors had no effect on the ramification or surveillance. Importantly, this research provided an interesting insight that directed extension and surveillance are distinct modes of microglial process dynamics and may be regulated by different molecular mechanisms.

Bernier et al. focused on the kinetics of filopodia growing from the main processes of microglia and observed that inhibition of actin polymerization by cytochalasin treatment stopped the extension and retraction of filopodia (9). Increasing intracellular cAMP concentrations by treatment with IBMX and norepinephrine reduced the number of large processes but increased the number of filopodia. Furthermore, activation of P2Y12 by ATP treatment abolished filopodia and promoted the elongation of large processes and the formation of bulbous tips. These results suggest that fluctuations in intracellular cAMP concentrations in the quiescent state lead to the formation of filopodia and random surveillance, whereas elevated extracellular ATP concentrations cause filopodia to retract, promoting unidirectional process extension and the formation of bulbous tips.

### Cultured Slice

When using acute slices, the effects of factors released due to the damage associated with section preparation need to be recognized. Cultured slices may partly overcome this point by recovering during the incubation period. In contrast to acute slices which require the experiment to be completed within 4 h of brain sectioning to observe ramified microglia (52), a major advantage of using cultured slices is the ability to image a relatively long time after manipulation, as it allows us to elucidate microglial changes in response to temporal changes (e.g., neurite outgrowth and transformation of the injured area). In addition, it is also possible to develop systems that recapitulate the synaptic connections between pre- and post-neurons by coculturing distant but neural-connected brain regions (e.g., retinal ganglion cells in retina and dorsal LGN neurons). However, one must be cautious of the unique reorganization of nerve fibers and glial proliferation in cultured slices and the different characteristics of microglia in the superficial and lower layers of slices (86). Additionally, as is often the case in culture systems, minute differences in techniques between experimenters of culture specimens may lead to significant differences in the morphology of microglia that are sensitive to environmental changes. The membrane used for culture sections is also important.

Recently, Ogaki et al. focused on the fact that PTEE membranes, which are frequently used to place slices in culture, make live imaging using inverted microscopy difficult due to the low light transmission of PTFE membranes (10) (Table 3). The authors evaluated the performance of the collagen membrane by placing sections on the light-permeable collagen membrane instead of the PTFE membrane and culturing the slices. The results showed that there was no significant difference in the number and viability of neurons and microglia between slices cultured on the PTFE membrane and slices cultured on the collagen membrane. In addition, the microglial processes, filopodia as well as cell contours were more clearly observed on the collagen membrane during live imaging. These innovations will broaden the use of slice culture for live imaging of microglia.

**Table 3:** Experimental conditions of live imaging of microglia *ex vivo* (slice culture).

**Animal**	**Age**	**Imaging timing**	**Region**	**Thickness ** **Depth from the surface**	**Cellular visualization**	**Microscopy**	**Objective lens**	**Resolution (x, y) ** **z stack**	**Interval time ** **Total time**	**References**
CX3CR1-GFP^+/−^ mice	P6-7	10–12 DIV	Hippocampus	300 μm	GFP (microglia)	Multi-photon	25x	– 21 planes with 1.5 μm step	1 min 15 min	Greenhalgh et al. (78)
CX3CR1-GFP^+/−^ mice	E14	–	Cortex	350 μm	GFP (microglia)	Confocal	–	–	30 min 8 h	Hattori et al. (79)
Iba1-EGFP^+/−^ mice	P6-7	13–14 DIV	Hippocampus	300 μm	GFP (microglia)	Fluorescence	20x (NA = 0.45)	–	20 min –	Katayama et al. (80)
Wistar rats	P7-9	10 DIV	Hippocampus	350 μm	CMTMR (microglia) CMAC (PMN)	Confocal	–	–	–	Neumann et al. (81)
CX3CR1-GFP^+/−^ mice	P6	7–9 DIV	Entorhinal-hippocampus	400 μm	GFP (microglia)	Confocal	30x (NA = 1.05)	– 21 planes with 1 μm step	30 s –	Ogaki et al. (10)
Iba1-EGFP^+/−^ mice	P4-7	–	Hippocampus	400 μm	GFP (microglia)	Confocal	20x	– 20 planes with 2 μm step	5 min 1 h	Ohsawa et al. (82)
SD rats	P4-6	1–8 DIV	Hippocampus	400 μm	IsolectinB4-FITC (microglia) Sytox Orange or To-Pro-3 (dead cell)	Confocal	20x (NA = 0.7)	512 × 512 pixel 5–10 planes with 5–8 μm step	2-4 min 2–11 h	Petersen et al. (83)
Cx3cr1::CreER; RC::LSL-tdTomato mice	P4	10–19 DIV	Hippocampus	300 μm 30 μm	tdTomato (microglia) neuron (iRFP)	Light sheet	60x (NA = 1)	0.13 × 0.13 μm 0.48 μm step	45–60 s 2–3 h	Weinhard et al. (84)
Rabbits	–	–	–	350 μm	Tomato Lectin 594 (microglia)	Confocal	20x	– 50–80 μm thick with 2–5 μm step	15 min 5.5–6 h	Zhang et al. (85)

*E, embryonic day; P, post-natal day; DIV, day in vitro; –, not available*.

#### Studies on Microglial Dynamics That Require Long-Term Observation

Ohsawa et al. examined the effect of ADP on microglial process extension; treatment with RGD, an integrin inhibitor, together with ADP bath application, inhibited process extension to ADP (82). In conjunction with other experiments, the authors indicated that activation of microglial P2Y12 by ATP, which increases the expression level of integrin β1 and promotes adhesion to the extracellular matrix, is important for microglial process extension.

Although microglia are known to repopulate once removed, the function and motility of repopulated microglia have not been well-elucidated. Zhang et al. performed live imaging of retinal explant cultures to examine the response to ATP of repopulated microglia (87). After 60 days of repopulation, there was no change in basal process motility or the degree of process elongation during ATP treatment between repopulated and endogenous microglia.

In the cortical plate (CP), microglia are temporarily absent from E15 to E16 and instead are abundant in the ventricular zone (VZ), subventricular zone (SVZ), and intermediate zone (IZ) (88). However, it is not clear why or how microglia are absent from the CP during this period: neurons arising in the VZ/SVZ migrate to the CP and divide into various subtypes corresponding to the cortical layer structure. Changes in the expression of transcription factors that control differentiation lead to abnormal layer structure and the emergence of ectopic neurons. Hattori et al. proposed and tested the hypothesis that microglia may be temporarily absent from the CP to prevent interference with the functional differentiation of CP neurons (79). Live imaging of slices prepared from E14 mice showed that microglia migrate from the CP to the meninges and that removal of the meninges reduced the amount of migration. A similar trend was observed using *in vivo* imaging *in utero*: contrary to the microglia in the CP, microglia in the VZ/SVZ/IZ were observed to migrate in the apical direction. In addition, microglial migration was inhibited in slices prepared from CXCR4-KO mice, indicating that the CXCL12-CXCR4 signaling pathway may regulate microglial migration. At present, verification of cell migration along the dorsoventral axis can only be performed by live *ex vivo* imaging, and this study demonstrates the utility of slice culture imaging.

#### Interactions Between Microglia and Other Cells

Multinucleated neutrophils (PMNs), which invade the brain parenchyma during ischemia, injure neurons by eliciting inflammation. Neumann et al. tested the possibility that microglia may play a role in protecting neurons by removing neutrophils by phagocytosis (81). Oxygen glucose deprivation (OGD) treatment of cultured slices increased neuronal death, which was further accelerated by the addition of PMNs. On the other hand, the addition of microglia at the same time as PMN reduced some of the neuronal death. Next, to test the possibility of microglial phagocytosis of PMNs, the authors added fluorescently labeled PMNs and microglia to cultured slices and performed live imaging. Both endogenous and added microglia phagocytosed PMNs. It was also shown that the microglia phagocytosed both dead and live PMNs. Furthermore, treatment with RGD and GluNAc (an inhibitor of lectins) reduced PMN phagocytosis and accelerated OGD treatment-induced neuronal death. These results suggest that phagocytosis of PMNs by microglia is neuroprotective.

Using live imaging of hippocampal culture slices, Weinhard et al. were able to capture the series of process in which microglia phagocytose the presynaptic structures (boutons) formed by dentate gyrus granule cells (84). They did not identify any instances of microglia phagocytosis of dendritic spines and argued that, at least under physiological conditions, microglia may preferentially phagocytose presynaptic components.

#### Studies on Brain Injury and Brain Diseases

In Petersen et al., the response of microglia to damaged neurons was observed at 1~7 DIV (83). When adjacent cells died, microglia extended their own processes and phagocytosed the dead cells. The motility of microglial processes changed after contact with other microglia and dead cells, suggesting that contact determines the target for phagocytosis.

Katayama et al. subjected neurons to excitotoxicity by NMDA and observed the response of microglia to injured neurons (80). Microglia accumulated in the pyramidal cell layer after injury, indicating that this was independent of microglial proliferation. In addition, PI-positive neurons disappeared after being surrounded by microglia, and this was inhibited by removal of the microglia by clodronate treatment, suggesting that microglia phagocytose the injured neurons. Furthermore, the authors showed that phagocytosis of PI-positive neurons was promoted by p38 MAP kinase.

Anti-inflammatory therapy that targets microglia to block the microglial inflammatory response in cerebral palsy and autism is currently being explored. Zhang et al. used *E. coli*-induced maternal immune activations (MIAs) in rabbit fetuses and prepared hemispheric slice cultures to examine microglial migration and the interaction between microglia and dendrimers (85). Microglia in the MIA group exhibited an amoeboid shape, and their migration speed and distance were reduced. Since the microglia in the MIA group took up more dendrimers at a faster time, the authors claimed that the change in microglial motility was due to an interaction with dendrimers and proposed that assays using culture slices could be useful for drug discovery.

Greenhalgh et al. examined the effect of bone marrow-derived macrophages on the responsiveness of microglia during tissue injury (78). Cultured slices were laser-irradiated, and the induction of microglial processes to the irradiated area was quantified. There was no change in each parameter of process extension in the presence or absence of macrophages.

## *In vitro* (Dispersed Cell Culture)

In section *Ex vivo* Live Imaging, live imaging of microglia using acute or cultured slices was introduced. Slice imaging has clearly broadened the scope of microglial studies, as it allows live imaging of the brain in its layered structure without area limitations. However, the heterogeneity of microglia depends on the position of the slice, which must be noted in slice imaging (86). Thus, most papers specify in the materials and methods section how much depth (Z-axis distance) the imaging was performed from the surface of the slice. It is important to have some degree of homogeneity in microglial conditions before treatment, especially when comparing microglial morphology and function between the control and treated groups. To overcome the heterogeneity of microglia, dispersed cultures would be useful. The greatest advantage of using dispersed cultures is that the cell types present in the observation system can be regulated according to the purpose of the experiment, which is naturally different from the *in vivo* conditions though. These features make it possible to clarify which cell type a particular phenomenon strongly depends on, and may facilitate the screening of the molecular mechanisms involved. Taking advantage of this, cell culture systems have been widely used for disease research, pharmacological screening, and, more recently, iPS microglia research. In the future, more sophisticated cell culture systems, especially in terms of reproducing *in vivo* microglial morphology, will be needed to validate cell-cell interactions that underlie neural circuitry and brain function.

The medium and presence of heterogeneous cells may have a significant impact on the morphology and functions of microglia. For example, Montilla et al. examined the effect of medium composition on microglial properties (93). Compared to DMEM+10% FBS, the use of TIC medium which contains TGF-β, IL-34, and cholesterol (8) resulted in an increase in microglial process number, changes in purine receptor expression levels, reduced phagocytic ability, and increased motility (movement speed and distance traveled), which indicated that serum significantly influences microglial conditions. There is also a significant influence of serum; Rizzi et al. examined the effect of NGF on the behavior of microglia (94), finding that microglial motility, which was defined as the degree of change in the ratio of cell area/cell perimeter length, was increased by NGF, although the cell body migration speed did not change.

### Disease Research

As mentioned above, the contribution of cell culture to disease research and drug screening systems is significant. Therefore, we present here a chronological list of some of the most important disease studies using live imaging of cell cultures. To mimic Huntington disease, microglia were cocultured with striatal and cortical neurons expressing Htt mutants (91) (Table 4). Live imaging showed that microglia moved into the vicinity of neuronal projections, which were then torn off. These results suggest that microglia cause neuronal degeneration.

**Table 4:** Experimental conditions of live imaging of microglia *in vitro*.

**Animal**	**Age**	**Isolation method**	**Region**	**Other cell type in the culture**	**Cellular visualization**	**Microscopy**	**Objective lens**	**Resolution (x, y) ** **z stack**	**Interval time ** **Total time**	**References**
SOD1-G93A mice (animal model of myotrophic lateral sclerosis)	4 mo	Density gradient centrifugation	Whole brain	Motor neuron	Red (microglia) GFP (motor neuron)	Confocal	–	–	–	Frakes et al. (89)
C57BL/6 mice	P3	Mild trypsinization of mix glial coculture	Cortex	Bacteria	DIC	Epifluorescence	10x or 20x	–	–	Hupp et al. (90)
mice or rats	P1-2	Shaking of mix glial coculture	Cortex	Neuron	IsolectinB4-594 (microglia) YFP (neuron)	–	–	–	–	Kraft et al. (91)
iPSCs	–	Differentiation	–	Synaptosome	DIC (microglia) pHrodo (synapptosome)	Confocal	–	0.61 × 0.61 μm –	–	Sellgren et al. (92)

*iPSCs, induced Pluripotent Stem Cells; P, post-natal day; mo, month-old; –, not available*.

To establish an *in vitro* model of amyotrophic lateral sclerosis (ALS), Frakes et al. cultured motor neurons from wild-type mice with microglia isolated by density gradient centrifugation from adult wild-type or SOD1-G93 mice (ALS model) (89). Seventy-two hours after the start of coculture, the number of motor neurons cocultured with microglia from the ALS model mice was reduced to ~50% of the number when cocultured with microglia from wild-type mice. When shRNA against the SOD1 mutant was expressed by lentiviruses to reduce SOD1 expression in microglia to ~25%, the reduction in motor neuron numbers was rescued. The authors also showed that the SOD1 mutant caused microglial inflammatory responses (increased expression of CD68, iNOS, and ROS) and motor neuron death via activation of NFκB. It should be noted that coculture with microglia did not affect neuronal death when microglia were isolated from neonatal microglia, suggesting that even isolated microglia may be able to reflect the condition of the derived individual to some extent.

In Hupp et al., the effect of *Streptococcus pneumoniae* on the function of microglia was examined (90). In both isolated cultures of microglia and cocultures with astrocytes, exposure to *Streptococcus pneumoniae* resulted in reduced chemotaxis to the bacteria and motility of microglia. This could be a strategy by which *Streptococcus pneumoniae* escapes phagocytosis by microglia.

Sellgren et al. developed an *in vitro* model of microglia-dependent synaptic removal to test the possibility that excessive synaptic removal may contribute to schizophrenia (92). The authors showed that synaptosomes derived from schizophrenia patients were more likely to be phagocytosed and that patient-derived microglia-like cells (iMGs) had a higher phagocytosis capacity. Furthermore, they found that C4, a schizophrenia risk gene, was involved in complement tagging to neurons and synaptic removal by microglia. Additionally, minocycline inhibited synaptic removal and reduced the risk of developing schizophrenia. Although representative images of differentiated iMGs showed a morphology relatively close to that *in vivo*, no image was presented to show the morphology of iMGs during synaptosome phagocytosis.

As reported by Hupp and Sellgren (see above), another advantage of the cell culture system is that the process of phagocytosis is easier to observe than other experimental systems. Taking advantage of this, Zhao et al. examined the effect of activation of mTOR signaling on phagocytosis in microglia (72). They added pHrodo to primary cultures of microglia and measured its uptake, finding that phagocytosis was enhanced in TSC1Cx3cr1CKO mice compared with microglia derived from wild-type mice. It should be noted that there are still no studies which succeeded in reproducing ramified morphology of microglia in the co-culture of microglia and neurons, and that *in vitro* system probably failed to reflect how microglia phagocyte neurons and synapses *in vivo*.

### Sophistication of Culture Systems

One of the most challenging aspects of microglial isolation and culture is recreating microglial morphology *in vivo*, and there have been many attempts to improve the morphology of microglia in culture. For example, it has been shown that ATP, which promotes microglial process extension, increases the number of microglial primary processes (95). Thus, it would be useful to target molecules that have already been shown to regulate microglial ramification *in vivo* and *ex vivo*.

It has also been shown that the method of coating the surface of the culture dish may affect microglial morphology (96, 97). A strong candidate for factors that promote microglial ramification are astrocyte-derived molecules; since the 1990s, coculturing microglia with astrocytes and adding astrocyte culture supernatants (ACMs) to isolated cultures of microglial conditions have been used to improve microglia *in vitro* (6, 98, 99). Recently, among astrocyte-derived factors, CSF-1, TGF-β, and cholesterol were found to be required for microglial ramification (8). This work was a breakthrough in microglial culture, as it allowed for studies to use *in vivo*-like microglia without coculturing with other cell types.

Because microglial functions, such as exploration of the brain environment and phagocytosis, are strongly dependent on the morphology of the process, recreating the ramified process is particularly important. Hyperramified microglia have been reported in the diseased brains of chronic despair models and alcoholism models (100, 101). Using electron microscopy, Bisht et al. also reported the existence of microglia with highly ramified processes, called dark microglia, in pathological conditions such as stress and Alzheimer's disease (102). A group of microglial genes whose expression levels are altered in each animal model is also being elucidated by RNA sequencing. Using this information, methods to promote microglial ramification *in vitro* may be discovered.

However, it should be noted that the ramified morphology of microglia *in vitro* does not necessarily reflect the characteristics of transcriptome under physiological conditions *in vivo*. Indeed, Bohlen et al. mentioned this limitation by comparing the RNA-sequence data between cultured microglia with ramified morphology and freshly isolated microglia (8).

## Conclusion and Perspective

In section Achievement and Limitation of *in vivo* Live Imaging, we described studies that have performed live imaging of microglia *in vivo, ex vivo* (acute slice and cultured slice), and *in vitro* (primary culture). Although we have not been able to cover all of the published papers, the number of papers is roughly in the order of *in vivo, ex vivo*, and *in vitro*. *In vitro* systems are easier than other experimental systems to perform pharmacological and genetic manipulations (e.g., induction of synaptic competition, which is important in neuron-microglial interactions). In addition, multicolor live imaging is essential to investigate the relationship between microglia and synapses. Since a synaptic site contains at least four components, i.e., microglia, astrocytes, pre-synapses, and post-synapses, all of these components should be imaged as simultaneously as possible at high resolution to assess the function and identity of “quadripartite synapses” (103–105). The pros of *in vitro* system is that multicolor live imaging can be accomplished without the need of complicated breeding schemes and crossings of transgenic animals to label multiple cellular elements.

However, live imaging systems using *in vitro* tools still have limitations that need to be solved. The most critical point is that *in vitro* microglia differ from *in vivo* and *ex vivo* microglia in that they exhibit an abnormal form (hypertrophy of the cell body and loss of fine processes). This microglial morphological abnormality is unsuitable for examining microglial-synaptic interactions, which have recently received considerable attention among microglial functions. Conversely, if we can establish an *in vitro* experimental system that overcomes the abnormal morphology, it would be a breakthrough that could greatly advance microglial research. In particular, in the case of microglial-synaptic interactions, the detailed molecular mechanisms cannot be explored without observing and manipulating cell-cell interactions in the glia-neural circuit complex. For example, other than microglia, the involvement of astrocytes cannot be disregarded. Astrocyte micro-processes surround pre-synapses and post-synapses, forming a tripartite synapse structure (106). The formation of tripartite synapses is then promoted by increased neural activity (107). Therefore, to elucidate the activity-dependent microglial-synaptic interaction, a four-party structure that adds microglia to the conventional tripartite synapse, or quadripartite synapse, should be studied. To do so, it is necessary to solve the difficult problem of morphological abnormalities in cultured microglia. If we can induce synaptic competition at the individual synapse level and subsequently detect changes in glial intracellular signaling and glial gene expression, we will obtain many novel insights into the synaptic plasticity that underlies brain function (Figure 2). In addition to morphological abnormalities, discrepancies of gene expression characteristics with *in vivo* microglia are also issues to be solved in *in vitro* experimental systems. In the future, it is expected to develop *in vitro* experimental systems that mimic the gene expression characteristics of microglia *in vivo*.

Another significant limitation of using *in vitro* systems may be the inability to fully reproduce the brain environment. There are many components of the brain that are lacking in cell culture and may affect microglial morphology and function, such as the blood brain barrier (BBB), blood vessels, extracellular matrix (ECM), and other cell types. In recent years, many attempts have been made to reconstitute BBB, blood vessels, and ECM *in vitro* (108, 109) and to culture ramified microglia with other cell types (110).

In conclusion, in order to understand the role of microglial dynamics in brain function and the underlying cellular and molecular mechanisms, it is important to develop appropriate *in vitro* live imaging systems that reflect the findings of *in vivo* live imaging and fully exploit the convenience of genetic and pharmacological manipulation of brain cells *in vitro*.

## Author Contributions

MA and RK wrote the manuscript. Both authors contributed to the article and approved the submitted version.

## Conflict of Interest

The authors declare that the research was conducted in the absence of any commercial or financial relationships that could be construed as a potential conflict of interest.
